# Development and assessment of a predictive nomogram for the progression of IgA nephropathy

**DOI:** 10.1038/s41598-018-25653-9

**Published:** 2018-05-09

**Authors:** Lin-lin Liu, Lin-bo Zhu, Jian-nan Zheng, Tong-dan Bi, Jian-fei Ma, Li-ning Wang, Li Yao

**Affiliations:** Department of Nephrology, The First Affiliated Hospital of China Medical University, Shen Yang Liao Ning, China

## Abstract

The present study is to establish a nomogram for predicting the prognosis of IgA nephropathy (IgAN). Of the 869 IgAN patients, four-fifths were randomly assigned to the development cohort and one-fifth to the validation cohort. The primary outcome was a composite event of either a ≥ 50% reduction in estimated glomerular filtration rate (eGFR), end-stage renal disease or death. The mean follow-up time was 44 months. The Cox regression model identified urinary protein excretion (1–3.5 g/d, HR 11.639, 95% CI 3.601–37.625; ≥ 3.5 g/d, HR 32.435, 95% CI 10.079–104.380), eGFR (G2, HR 5.293, 95% CI 2.011–13.932; G3, HR 15.797, 95% CI 6.584–37.905; G4, HR 34.619, 95% CI 13.887–86.301; G5, HR 217.651, 95% CI 83.807–565.248), hyperuricaemia (HR 7.031, 95% CI 4.126–11.980), mesangial proliferation (HR 36.667, 95% CI 5.098–263.711), segmental glomerulosclerosis (HR 5.122, 95% CI 3.114–8.425), tubular atrophy/interstitial fibrosis (T1, HR 33.351, 95% CI 7.831–142.044; T2, HR 213.888, 95% CI 51.048–896.182), crescents (C1, HR 3.123, 95% CI 1.771–5.510; C2, HR 7.353, 95% CI 3.590–15.062) and glomerulosclerosis (25–49%, HR 3.123, 95% CI 1.771–5.510; ≥ 50%, HR 14.384, 95% CI 8.813–23.479) for developing the nomogram. The C-index was 0.945 (95% CI 0.914–0.976) in both the development and validation cohorts, showing good agreement between the nomogram-predicted probability and actual free-of-progression probability. Thus, our nomogram could accurately predict the progression of IgAN patients.

## Introduction

IgA nephropathy (IgAN) is one of the most prevalent forms of primary glomerulonephritis worldwide^1^. IgAN can develop at any age but appears particularly common in young adults. The clinical course of IgAN is highly variable, ranging from asymptomatic haematuria/proteinuria and nephrotic syndrome to acute progressive glomerulonephritis. Correspondingly, the pathological features also present multiple forms, such as active inflammatory renal pathology features (i.e., crescentic formation) and chronic glomerular inflammation, which leads to mesangial proliferation, segmental glomerulosclerosis and tubular-interstitial fibrosis.

IgAN is not a benign condition; it is an important cause of end-stage renal disease (ESRD), thereby representing a major health challenge worldwide^2^. The majority of IgAN patients have a progressive disease course, with 10–30% of cases reaching ESRD within 10 years after diagnosis^1–3^. The most recent STOP-IgAN trial showed no benefit of immunosuppression for “high-risk” patients with IgAN^4^. In fact, the definition of “high-risk” remains uncertain. There is an urgent need for a strategy to identify IgAN patients at high risk for poor prognosis; this strategy may help to determine treatment regimens and predict the prognosis of IgAN. During the past two decades, some clinical, pathological and genetic parameters have been identified as markers for predicting the progression of IgAN^5–8^. However, the value of a single marker is limited, and different markers have various contributions to prediction. Therefore, building a method with multiple useful markers with consideration of their contribution to an accurate prediction of the prognosis of IgAN patients is necessary.

Nomogram, a statistics-based tool that provides the overall probability of a specific outcome, has been widely used in many diseases^9–11^ and can effectively and visually predict the progression of a disease. Currently, to our knowledge, no ideal nomogram has been established to predict the prognosis of IgAN patients. Thus, the aim of this study is to establish a useful predictive model to accurately identify the prognosis of IgAN patients.

## Results

### Clinicopathological characteristics of the included patients

The present study included 869 IgAN patients with complete data. The demographic and clinicopathological characteristics of the 869 patients are presented in Table 1. In the primary cohort, 50.7% of the patients were male. The median age was 34 years old (range 14–77), and the majority of patients were less than 40 years old. Among the patients, 29.3% had a history of chronic tonsillitis, 37.9% developed hypertension at the start of the study, and 43.6% had hyperuricaemia. Only 18.9% of patients presented with proteinuria in the nephrotic range. The mean urinary protein excretion (UPE) and eGFR levels were 2.22 ± 2.44 g/d and 84.16 ± 35.25 ml/min/1.73 m^2^, respectively. The majority of patients had normal levels of IgA, complement C3 and complement C4. Regarding the pathological parameters, 77.7% of patients presented with significant mesangial hypercellularity (M1), 47.8% with significant segmental glomerulosclerosis (S1), and 25.1% with significant endocapillary hypercellularity (E1). More than half of the patients had no significant tubular atrophy or interstitial fibrosis (T0) and no crescent (C0). Furthermore, 69% of patients had no significant glomerulosclerosis (<25% of glomeruli). With respect to the therapeutic regimens, the majority of patients received renin angiotensin system inhibitors (RASI) (69.4%) and immunosuppressants (73.8%). The mean follow-up time was 44 ± 20 months. The incidence of the composite endpoint of either a ≥50% reduction in eGFR or ESRD or death was 13.3% in the primary cohort. There were no significantly different variables between the development and validation cohorts (all P > 0.05).

**Table 1:** Demographic and clinicopathological variables of the primary, development and validation cohorts.

	Primary cohort Number (%)	Development cohort Number (%)	Validation cohort Number (%)	P value
**Serum C4**				0.903
Low	77 (8.9)	61 (8.8)	16 (9.2)	
Normal	743 (85.5)	593 (85.2)	150 (8.7)	
High	49 (5.6)	42 (6.0)	7 (4.0)	
**Oxford classification**				
M0	194 (22.3)	153 (22.0)	41 (23.7)	0.889
M1	675 (77.7)	543 (78.0)	132 (76.3)	
E0	651 (74.9)	525 (75.4)	126 (72.8)	0.780
E1	218 (25.1)	171 (24.6)	47 (27.2)	
S0	454 (52.2)	367 (52.7)	87 (50.3)	0.848
S1	415 (47.8)	329 (47.3)	86 (49.7)	
T0	496 (57.1)	402 (57.8)	94 (54.3)	0.915
T1	227 (26.1)	181 (26.0)	46 (26.6)	
T2	146 (16.8)	113 (16.2)	33 (19.1)	
C0	471 (54.2)	389 (55.9)	82 (47.4)	0.235
C1	331 (38.1)	259 (37.2)	72 (41.6)	
C2	67 (7.7)	48 (6.9)	19 (11.0)	
**Glomerulosclerosis**				0.999
<25%	600 (69.0)	479 (68.8)	121 (69.9)	
25–49%	178 (20.5)	144 (20.7)	34 (19.7)	
≥50%	91 (10.5)	73 (10.5)	18 (10.4)	
**RASI**				0.931
Not received	266 (30.6)	211 (30.3)	55 (31.8)	
Received	603 (69.4)	485 (69.7)	118 (68.2)	
**Immunosuppressants**				0.993
Not received	228 (26.2)	182 (26.1)	46 (26.6)	
Received	641 (73.8)	514 (73.9)	127 (73.4)	
**Composite endpoint**				0.472
No	753 (86.7)	608 (87.4)	145 (83.8)	
Yes	116 (13.3)	88 (12.6)	28 (16.2)	

UPE, urinary protein excretion; eGFR, estimated glomerular filtration rate; RASI, renin-angiotensin system inhibitors.

### Nomogram development

Univariate analysis showed that the composite endpoint was significantly associated with sex, hypertension, UPE, eGFR, hyperuricaemia, serum levels of complement C4, mesangial hypercellularity, endocapillary hypercellularity, segmental sclerosis, tubular atrophy or interstitial fibrosis, crescents, glomerulosclerosis and administration of RASI. The composite endpoint was not associated with age, history of chronic tonsillitis, serum levels of IgA and complement C3 and administration of immunosuppressants (Table 2). Furthermore, multivariate analysis identified UPE, eGFR, hyperuricaemia, tubular atrophy/interstitial fibrosis, crescents and glomerulosclerosis as independent predictive factors for the composite endpoint (Table 2).

**Table 2:** Univariate and multivariable Cox proportional hazards regression analysis of the data from the development cohort.

Predictors	Univariable analysis			Multivariable analysis		
	HR	95% CI	P value	HR	95% CI	P value
**Sex**			0.024			0.629
Male	1.000			1.000		
female	0.609	0.395, 0.938	0.024	1.122	0.704, 1.787	0.629
**Age at renal biopsy**			0.158	—	—	—
<40	1.000					
40–59	1.661	1.027, 2.688	0.039			
50–59	1.261	0.636, 2.499	0.507			
60–69	1.776	0.638, 4.945	0.272			
≥70	3.185	0.771, 13.154	0.109			
**Chronic tonsillitis**			0.176	—	—	—
No	1.000					
Yes	0.703	0.423, 1.170	0.176			
**Hypertension**			<0.001			0.694
No	1.000			1.000		
Yes	3.958	2.534, 6.182	<0.001	1.111	0.656, 1.882	0.694
**UPE (g/d)**			<0.001			0.028
<1				1.000		
1–3.5	11.639	3.601, 37.625	<0.001	1.928	0.566, 6.563	0.294
≥3.5	32.435	10.079, 104.380	<0.001	3.347	0.974, 11.501	0.055
**eGFR (ml/min/1.73 m** ^**2**^ **)**			<0.001			<0.001
G1	1.000			1.000		
G2	5.293	2.011, 13.932	0.001	0.940	0.321, 2.754	0.911
G3	15.797	6.584, 37.905	<0.001	1.045	0.381, 2.866	0.932
G4	34.619	13.887, 86.301	<0.001	2.086	0.673, 6.466	0.203
G5	217.651	83.807, 565.248	<0.001	9.871	2.845, 34.250	<0.001
**Hyperuricemia**			<0.001			0.024
No	1.000			1.000		
Yes	7.031	4.126, 11.980	<0.001	1.970	1.095, 3.543	0.024
**Serum IgA**			0.562	—	—	—
Normal	1.000					
High	0.867	0.535, 1.406	0.562			
**Serum C3**			0.060	—	—	—
Low	1.000					
Normal	1.817	0.735, 4.491	0.196			
High	4.382	1.266, 15.166	0.020			
**Serum C4**			<0.001			0.061
Low	1.000			1.000		
Normal	8.008	1.113, 57.629	0.039	2.086	0.277, 15.706	0.475
High	26.498	3.480, 201.788	0.002	4.51	0.551, 36.955	0.160
**Oxford classification**						
**Mesangial hypercellularity**			<0.001			0.092
M0	1.000			1.000		
M1	36.667	5.098, 263.711	<0.001	5.639	0.752, 42.273	0.092
**Endocapillary hypercellularity**			0.023			0.553
E0	1.000			1.000		
E1	1.656	1.072, 2.557	0.023	1.164	0.705, 1.919	0.553
**Segmental sclerosis**			<0.001			0.179
S0	1.000					
S1	5.122	3.114, 8.425	<0.001	1.458	0.841, 2.526	0.179
**Tubular atrophy/interstitial fibrosis**			<0.001			<0.001
T0	1.000			1.000		
T1	33.351	7.831, 142.044	<0.001	9.322	1.981, 43.873	0.005
T2	213.888	51.048, 896.182	<0.001	24.049	4.648, 116.917	<0.001
**Crescents**			<0.001			<0.001
C0	1.000			1.000		
C1	4.433	2.677, 7.342	<0.001	2.262	1.295, 3.950	0.004
C2	7.353	3.590, 15.062	<0.0001	5.207	2.290, 11.838	<0.001
**Glomerulosclerosis**			<0.001			0.033
<25%	1.000			1.000		
25–49%	3.123	1.771, 5.510	<0.001	0.897	0.470, 1.711	0.742
≥50%	14.384	8.813, 23.479	<0.001	2.023	1.097, 3.729	0.024
**RASI**			<0.001			0.589
Not received	1.000			1.000		
Received	0.357	0.234, 0.545	<0.001	1.158	0.680, 1.973	0.589
**Immunosuppressants**			0.328	—	—	—
Not received	1.000					
Received	1.296	0.771, 2.177	0.328			

UPE, urinary protein excretion; eGFR, estimated glomerular filtration rate; RASI, renin-angiotensin system inhibitors.

**Figure 1:**
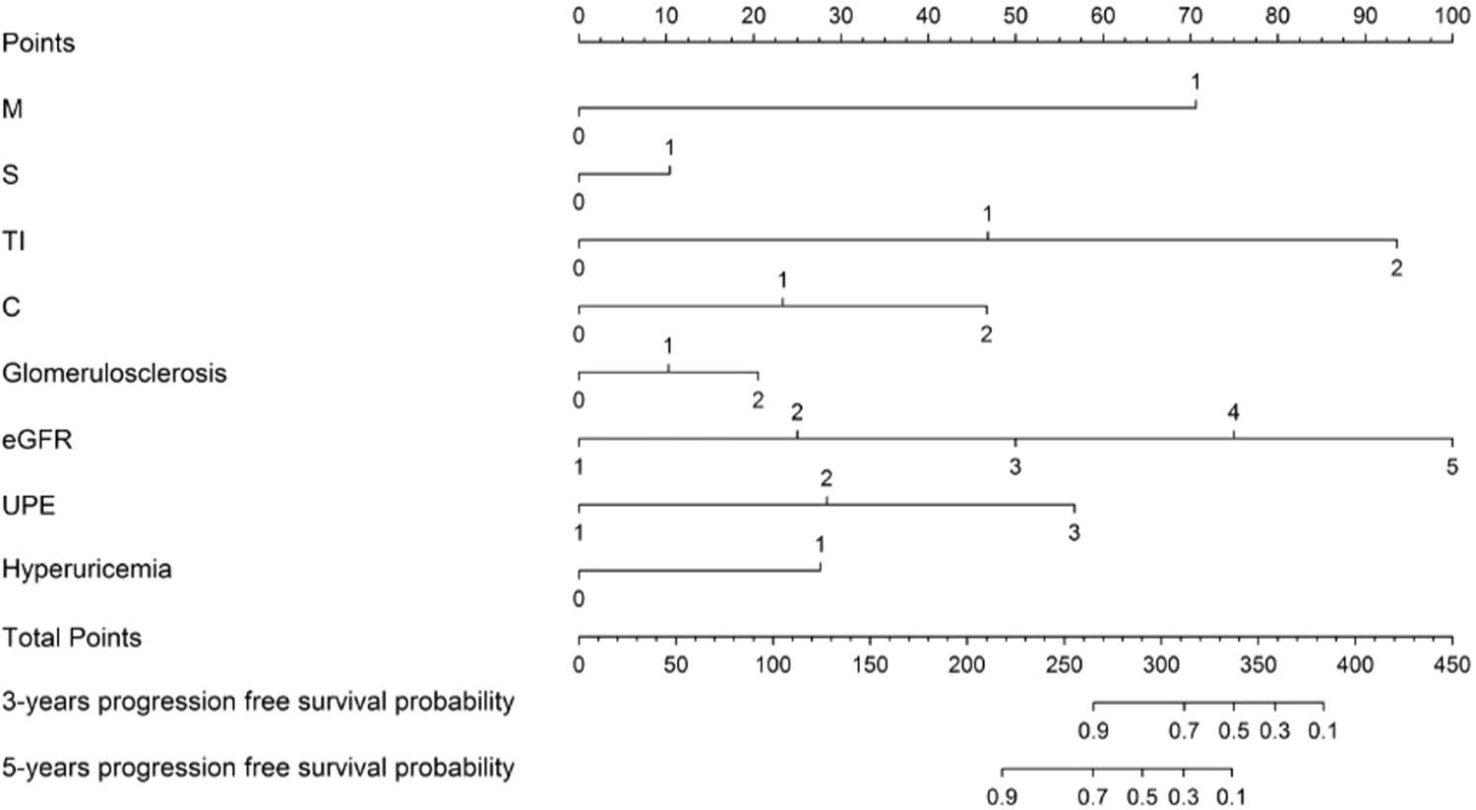
Nomogram for predicting the 3- and 5-year prognosis of IgAN patients. The nomogram was applied by summing the points identified on the points scale for each variable. According to the total points on the bottom scales, the nomogram provides the probability of the 3- and 5-year prognosis for an individual patient. Abbreviation: M, mesangial hypercellularity; E, endocapillary hypercellularity; S, segmental glomerulosclerosis, TI, tubular atrophy/interstitial fibrosis, C, crescents; eGFR, estimated glomerular filtration rate; UPE, urinary protein excretion.

According to the results of multivariate analysis, we included UPE, eGFR, hyperuricaemia, tubular atrophy/interstitial fibrosis, crescents and glomerulosclerosis to build a nomogram for predicting the 3- and 5-year prognosis of IgAN patients. In addition, although our analysis did not identify mesangial hypercellularity and segmental sclerosis as independent predictive factors, we included these variables in the nomogram according to the Oxford classification recommendations^5^. We did not include endocapillary hypercellularity since many studies have failed to show its prognostic value^12–14^. For each predictive factor in the nomogram, the point was read out by drawing a line straight upward from each predictor to the point axis. The total point was calculated by summing each point located in the total point axis, which was further converted to probability (see the bottom scale). The concordance index (C-index) for the developed nomogram was 0.945 (95% CI 0.913–0.976). The calibration curves showed good agreement between the nomogram-predicted progression probability and the actual progression probability (Fig. 1).

### Nomogram validation

**Figure 2:**
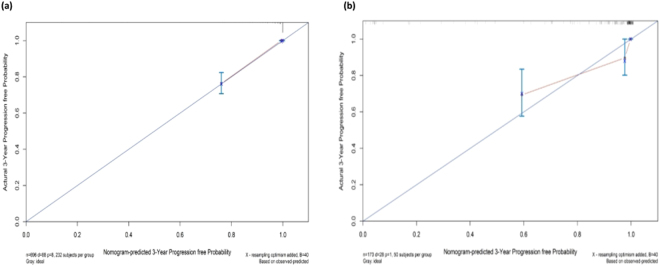
The calibration curves of the nomogram for predicting the 3-year prognosis of IgAN patients in the development cohort (**a**) and validation cohort (**b**). The X-axis represents the nomogram-predicted probability of progression, and the Y-axis represents the actual probability estimated with the Kaplan-Meier method. The blue line represents the ideal correlation between nomogram-predicted and actual probability.

**Figure 3:**
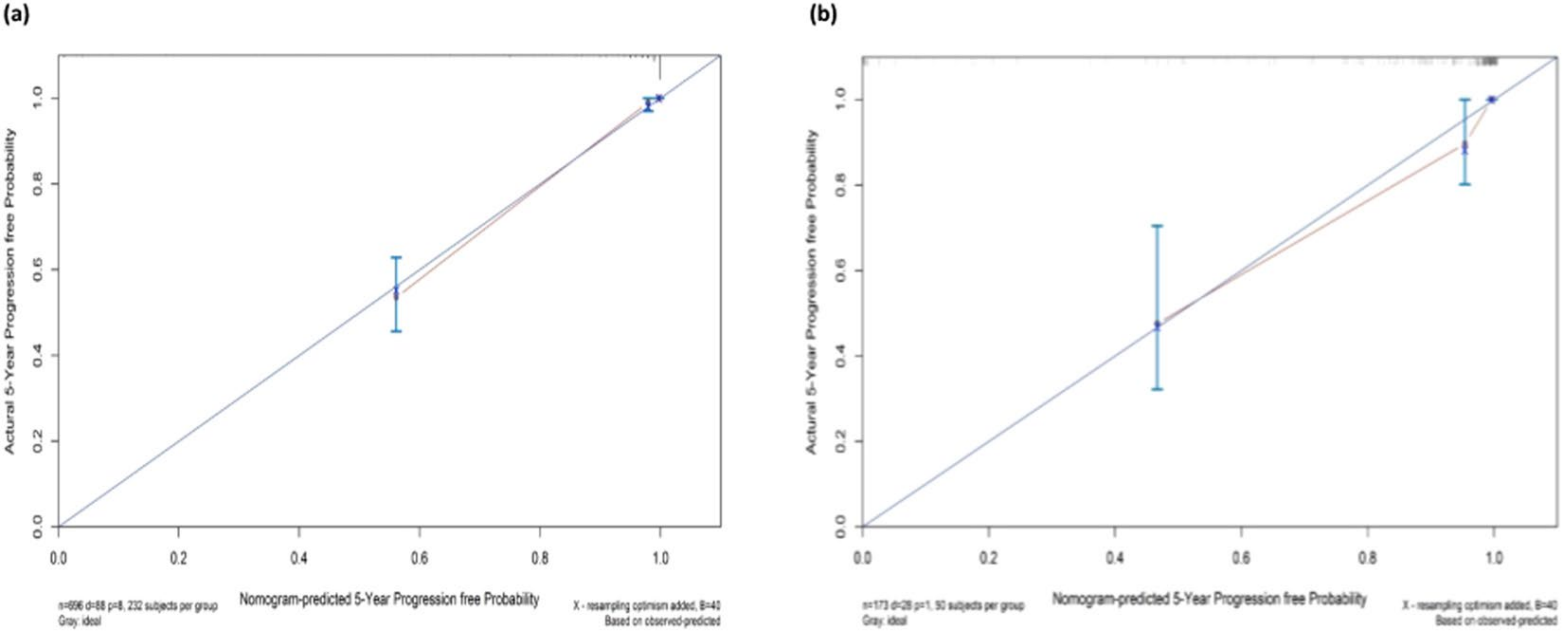
The calibration curves of the nomogram for predicting the 5-year prognosis of IgAN patients in the development cohort (**a**) and validation cohort (**b**). The X-axis represents the nomogram-predicted probability of progression, and the Y-axis represents the actual probability estimated with the Kaplan-Meier method. The blue line represents the ideal correlation between nomogram-predicted and actual probability.

In the validation cohort, the C-index for predicting the composite endpoint remained 0.945 (95% CI 0.914–0.976), and the calibration curves also showed our nomogram performing well in predicting the 3- and 5-year prognosis of IgAN patients (Figs 2,3).

**Figure 4:**
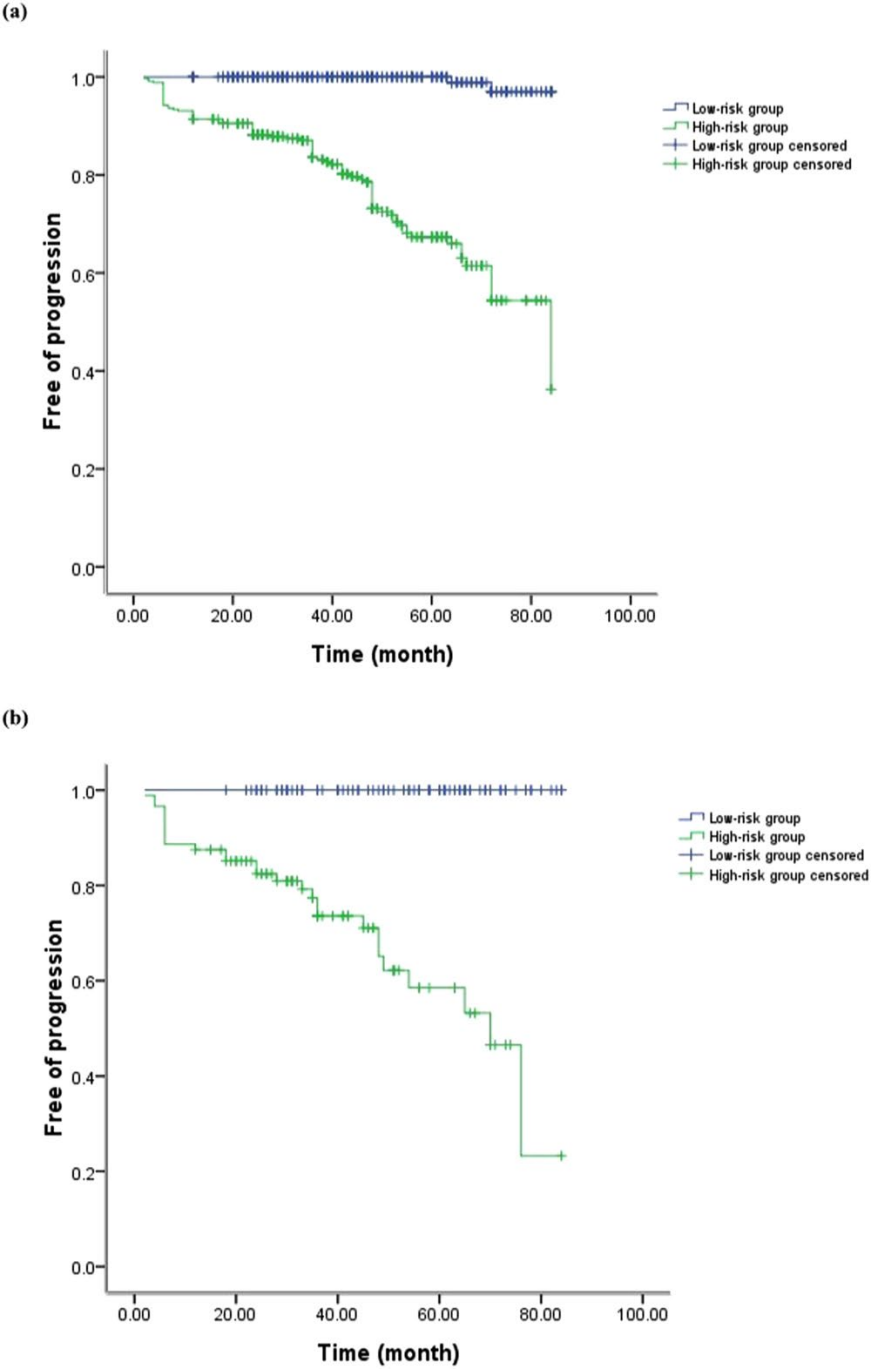
Kaplan-Meier survival curves of the development cohort (**a**) and the validation cohort (**b**) according to risk stratification of predicted free-of-progression probability.

Furthermore, we evaluated the discrimination ability of the nomogram by dividing the patients into two groups, specifically, low-risk and high-risk groups according to the points and plotted the Kaplan-Meier curves of both the development and validation cohorts (Fig. 4). The survival curves confirmed that the nomogram had good discriminatory ability for the patients.

## Discussion

In the present study, we included 869 IgAN patients and selected variables according to the results of the Cox proportional hazards regression model and the recommendation of the Oxford classification. Furthermore, we developed and validated the nomogram with C-indices and calibration curves. The C-indices of the development and validation cohorts were both 0.945, and all the calibration curves showed good agreement between the nomogram-predicted progression probability and the actual progression probability. The results of the Kaplan-Meier curves showed that our nomogram had good discriminatory ability for patients with low or high risk.

IgAN is a heterogeneous disease, and predicting the prognosis of individual IgAN patients is challenging. When clinical and pathological predictors are considered in isolation, they are inaccurate and even unreliable for evaluating the risk of progression of IgAN. Thus, there is an urgent need to build a comprehensive risk prediction model by combining these predictors. Until now, no ideal risk prediction model has been sufficiently validated for predicting the progression of IgAN. Yuan *et al.*^15^ developed a nomogram with the data of 489 patients for predicting the relapse of IgAN. Only two clinical parameters, gender and DBP, were included, and the C-index was 0.78. However, this study did not investigate the hard endpoint of renal outcome. Another study by Liu *et al.*^16^ included 349 IgAN patients and 4 variables (i.e., mesangial cell proliferation, tubulointerstitial lesions, proteinuria and mean arterial pressure) and developed a nomogram with a C-index of 0.88. However, the small sample size and relatively low C-index may limit the clinical utility of the nomogram. As such, no nomograms are currently accepted for clinical use. Compared with previous research, our nomogram with a larger sample size and higher C-index may supply more important clues in predicting individualized prognosis in IgAN patients and may be more applicable for clinical practice.

In the present nomogram, we first included UPE, eGFR, hyperuricaemia, tubular atrophy/interstitial fibrosis, crescents and glomerulosclerosis according to the results of the Cox proportional hazards regression model. These variables were also identified as independent risk factors in previous studies^5,17–19^. Notably, the Cox proportional hazards regression model did not identify mesangial hypercellularity and segmental sclerosis as predictors, but we selected them because they were recommended by the Oxford classification^5^ and verified by replication studies^12–14^. We attributed this difference to the discrepancy in sample sizes. In addition, hypertension was not identified as an independent risk factor based on our analysis, which may be imputed to the administration of RASI neutralizing the effect of hypertension for the prognosis of IgAN.

The present study has some potential limitations. First, the nomogram was developed and validated based on a retrospective analysis of the clinicopathological data from a single institution, which may bias our conclusions. Before it can be extensively applied, our nomogram requires further evaluation by external validation using data from multiple centres. Second, the time span of this study is large (from 2010 to 2015), which may lead to external variations in long-term prognosis^20^. Finally, the patients included in the present study received different treatment regimens, which may create unavoidable biases in the results. Future research should be based on data from multiple centres worldwide to develop and externally validate an ideal risk prediction model. The clinical and pathological predictors included in this model should be reliable and convenient for clinical application; moreover, they are expected to be the gold-standard for the risk evaluation of IgAN.

In recent years, in addition to clinical and pathological predictors, noninvasive biomarkers have attracted considerable attention; for example, gd-IgA1^21,22^ and some inflammatory markers^23,24^ may be involved in the immunopathogenesis of IgAN and have predictive value for the prognosis and activity of IgAN. These markers may be more sensitive for detecting the progression of early renal lesions in IgAN. Novel technologic and analytic approaches will aid in identification of noninvasive markers of the activity and prognosis of IgAN.

In conclusion, the nomogram developed in the present study is based on important clinicopathological predictors and could accurately predict the progression of IgAN patients. We supplied a novel and practical method to evaluate the prognosis of IgAN patients. However, the predictive value of our nomogram must be further verified externally in other institutions. Future research should be directed towards building an ideal risk prediction model by combining clinicopathological predictors and noninvasive biomarkers based on international multiethnic data.

## Materials and Methods

### Patients

We included patients according to the following criteria: (1) diagnosed with IgAN by renal biopsy in our department from January 2010 to December 2015; (2) 14 years of age or older; and (3) complete data, which were necessary for developing the nomogram.

Patients were excluded for the following reasons: (1) missing data, (2) treated with corticosteroids and immunosuppressants before the start of the study, (3) follow-up periods of less than one year, and (4) secondary IgAN. Causes of secondary IgAN included systemic lupus erythaematosus, Henoch-Schonlein purpura, ankylosing spondylitis, psoriasis, and liver disease.

The start day of the study was defined as the day of renal biopsy. The follow-up was performed until December 2016. Patients with renal dysfunction were routinely evaluated as “acute” or “chronic” according to their conditions in the last 3 months of follow-up. Patients with chronic renal dysfunction were excluded from renal biopsy except when they experienced acute exacerbation. Informed consent was obtained from all patients before renal biopsy. This study adhered to the recommendations outlined in the Declaration of Helsinki Principles and was approved by the Ethics Committee and the Research Board of our institution.

### Treatment

In our department, the initial therapeutic regimens for IgAN were applied as follows: (1) patients with haematuria only or haematuria combined with proteinuria less than 1 g/24 h and normal renal function were administered a non-immunosuppressive therapeutic regimen [e.g., angiotensin converting enzyme inhibitors (ACEI) or angiotensin II receptor blockers (ARB), fish oil, statins and anti-platelets]; and (2) patients presenting with persistent proteinuria of 1.0 g/24 h or more and active inflammatory pathological manifestations of cellular/fibrocellular crescents, moderate to severe mesangial proliferation and/or interstitial cell infiltration were administered immunosuppressive regimens, including corticosteroids, cyclophosphamide, mycophenolate mofetil, leflunomide or tripterygium glycosides, which were administered alone or in combination. Non-immunosuppressive regimens were also applied when needed.

### Data extraction

The following data were extracted from patients’ records: gender; age at diagnosis; histories of hypertension and overt haematuria; serum levels of creatinine, albumin, haemoglobin, uric acid, low-density lipoprotein cholesterol (LDL-C), high-density lipoprotein cholesterol (HDL-C), triglyceride, complement C3, complement C4, IgG, IgA and IgM; routine urinalysis; 24-h UPE; and therapeutic regimens.

During follow-up, serum creatinine, UPE, blood pressure and therapeutic regimen data were also collected. eGFRs were calculated by the Chronic Kidney Disease Epidemiology Collaboration (CKD-EPI) formula^25^.

### Renal pathological evaluation

A minimum of 8 glomeruli is necessary for diagnostics. Renal tissue sections were stained with haematoxylin-eosin, periodic acid-Schiff, periodic acid-silver methenamine and Masson’s trichrome for light microscopy. The tissue was stained with antibodies against IgG, IgA, IgM, C3, C1q and fibrinogen for immunofluorescence. Two pathologists performed individual renal pathological evaluations. According to the updated Oxford classification^5^, we evaluated the following five pathological parameters: mesangial proliferation, segmental glomerulosclerosis, endocapillary hypercellularity, tubular atrophy/interstitial fibrosis and cellular and/or fibrocellular crescents. In addition, we calculated the percentage of glomerulosclerosis and the presence of arteriolar lesions manifested by intimal thickening, hyaline degeneration and vessel lumen reduction. Disagreements between the two pathologists were resolved by discussion and consensus.

### Definitions

The primary outcome in this study was defined as a composite event of either a ≥50% reduction in eGFR, ESRD or death. ESRD was diagnosed if eGFR was less than 15 ml/min/1.73 m^2^ or renal replacement therapy, including haemodialysis, peritoneal dialysis or renal transplantation, was initiated. Hypertension was diagnosed if arterial blood pressures in resting states were at or above 140/90 mmHg no less than twice on different days after hospitalization (ambulatory blood pressure monitoring or measurement at irregular intervals) or if anti-hypertensive medications were administered to reach the target levels of less than 140/90 mmHg.

### Statistical analyses

Categorical variables are expressed with absolute frequencies and percentages, and normally distributed continuous variables are expressed as the means ± standard deviation or median with quartile range. The nomogram assumes a linear correlation between the predictive factors and patient prognosis^26^. Restricted cubic splines were applied to evaluate the linear correlation between continuous variables and prognosis. Before building the model, continuous variables were transformed into categorical variables to fit the linear assumption. Renal function was categorized into 5 grades according to the levels of eGFR as follows: grade 1 was defined as eGFR ≥ 90 ml/min/1.73 m^2^, grade 2 as eGFR of 60–89 ml/min/1.73 m^2^, grade 3 as eGFR of 30–59 ml/min/1.73 m^2^, grade 4 as eGFR of 15–29 ml/min/1.73 m^2^, and grade 5 as eGFR of less than 15 ml/min/1.73 m^2^. The levels of UPE were categorized into 3 grades as follows: <1 g/d, 1–3.49 g/d and ≥3.5 g/d. The ratios of glomerulosclerosis were also classified into 3 grades as follows: <25%, 25–49% and ≥50%.

Nomogram construction was performed according to the guidelines proposed by Iasonos^27^. We randomly assigned four-fifths of patients to the development cohort (n = 696) and one-fifth of patients to the validation cohort (n = 173).

To identify independent prognostic predictors, we used a Cox proportional hazards regression model for univariable and multivariable analyses by the “Enter” method. The nomogram was developed to predict the 3- and 5-year prognosis mainly based on the results of the multivariable Cox regression model.

The performance of the nomogram was estimated regarding discrimination and calibration. The C-index was applied to evaluate discrimination^26^, which refers to the models’ ability to accurately distinguish the outcomes. A higher C-index indicates more precise model predictions^28^. Calibration curves were performed by comparing the means of the nomogram-predicted outcomes with the actual outcomes estimated with Kaplan-Meier. The bootstrapping (1000 repetitions) method was applied to reduce the estimate bias. In addition, model validations were performed using the data of the validation cohort as follows. First, we calculated the total points of the patients in the validation cohort using the established nomogram. Next, we used the total points as a factor to perform Cox regression analysis. Finally, the C-index and calibration curves were developed with the results of regression analysis.

Statistical analyses were performed with SPSS version 22.0 (SPSS, Inc., Chicago, IL, USA) and R 3.4.1 (The R Foundation for Statistical Computing, Vienna, Austria). P values < 0.05 were considered statistically significant.
